# Pain Self-efficacy Is Associated With Patient-Reported Function in Individuals With Chronic Hip Pain

**DOI:** 10.2519/josptopen.2024.1021

**Published:** 2024-06-06

**Authors:** Nicholas C. Coyne, Shelby Baez, Millissia Murro, Demitria Derrico, Corrie A. Mancinelli, Kate N. Jochimsen

**Affiliations:** 1 School of Medicine, West Virginia University, Morgantown, WV; 2 Exercise and Sport Science, College of Arts and Sciences, University of North Carolina at Chapel Hill, Chapel Hill, NC; 3 Gait Biomechanics Laboratory, University of Delaware, Newark, DE; 4 Athletic Training Services, Boston University, Boston, MA; 5 Center for Health Outcomes and Interdisciplinary Research (CHOIR), Harvard Medical School, Boston, MA

**Keywords:** arthritis (OA), hip, outcome measures, pain, psychology, tendinopathy

## Abstract

**OBJECTIVE:**

To evaluate the associations between psychological factors (pain self-efficacy, kinesiophobia, and pain catastrophizing), physical activity, and patient-reported hip function in patients presenting to physical therapy with chronic (>3 months) hip pain.

**DESIGN:**

Observational, cross-sectional.

**METHODS:**

Participants completed a survey including age, sex, height/weight, symptom duration, 11-item Tampa Scale for Kinesiophobia (TSK-11), Pain Catastrophizing Scale (PCS), Pain Self-Efficacy Questionnaire (PSEQ), and 12-item International Hip Outcome Tool (iHOT-12). Participants wore an accelerometer (60 Hz) for 7 days. Predictors of iHOT-12 scores were assessed using a linear regression with forward variable selection.

**RESULTS:**

Forty-one participants (29 females, 12 males; 40.5 ± 14.0 years; 26.7 ± 7.8 kg/m^2^) with intra-articular nonarthritic hip condition (53.7%), hip osteoarthritis (19.5%), other/multiple diagnoses (17.1%), and extra-articular hip condition (9.8%) were evaluated. Diagnosis groups did not differ in sex, body mass index, physical activity, psychological measures, or patient-reported function (*P*≥.09). Participants with hip osteoarthritis (59.8 ± 8.3 years) were older than those with intra-articular nonarthritic hip conditions (33.0 ± 9.7 years) and other/multiple diagnoses (37.4 ± 10.6) (*P*<.001). A model containing PSEQ scores, moderate-to-vigorous physical activity, and TSK-11 scores explained 38% of the variance in iHOT-12 scores (*P*<.001), with PSEQ explaining 20% of the variance in iHOT-12 scores alone.

**CONCLUSION:**

Pain self-efficacy and kinesiophobia were associated with patient-reported function in people with chronic hip pain of multiple etiologies. Clinicians may consider screening for psychological factors in this patient population.

Musculoskeletal pain is highly prevalent and associated with high personal burden including functional disability and medical expenses.^3^ Among US adults aged 18 years or older, it has been estimated that more than 1 in 2 adults experiences pain from a chronic musculoskeletal disorder.^34^ Regular exercise regimens reduce chronic musculoskeletal pain through a variety of mechanisms, including weight management, muscle strengthening and optimal joint loading, distraction/shifting the focus and away from the pain, stress reduction, release of endorphins.^29^ However, individuals with chronic musculoskeletal pain (eg, low back pain) often reduce their activity in an attempt to manage their symptoms.^37^ Specifically, people with chronic hip pain conditions (eg, greater trochanteric pain syndrome) are usually less active than those without pain, and current interventions may not succeed in restoring optimal physical activity and function.^8,23,25,38,47^

In the biopsychosocial model of chronic pain, psychological factors can play an important role in the experience of pain.^12,30^ Mainly, kinesiophobia, pain catastrophizing, and self-efficacy beliefs have been widely studied in people with chronic musculoskeletal pain.^26,28,45^ Pain self-efficacy describes the personal confidence to cope with pain and participate in valued daily activities despite pain.^26,44^ Kinesiophobia (ie, fear of painful movement/reinjury) and pain catastrophizing (ie, worst-case thinking) are included in the fear-avoidance model (FAM) of chronic pain, which posits individuals with high levels of kinesiophobia and pain catastrophizing are more likely to adopt avoidance behaviors, which may increase the risk of disability and disuse.^46^ Self-efficacy is also often included in the FAM as it plays an important mediating role in perpetuating avoidance behavior.^49^ In this sense, pain catastrophizing, kinesiophobia, and pain self-efficacy will be selected for study. Additionally, these psychological factors are commonly assessed in chronic hip pain literature, allowing for contextualizing this study’s findings.

In individuals with chronic hip pain, maladaptive pain-related thoughts and feelings have been associated with worse pain, function, and less improvement following intervention.^16,20,22,38^ Following the FAM, improving pain-related thoughts and feelings may be associated with decreased avoidance (eg, increased physical activity) and improved pain and functional outcomes for patients with chronic hip pain. However, it is unclear which of these factors, individually or in combination, are most significantly associated with patient-reported function for patients attending physical therapy. The purpose of this study was to evaluate the associations between kinesiophobia, pain catastrophizing, and pain self-efficacy with physical activity and patient-reported hip function in people with chronic hip pain attending physical therapy. We hypothesized that better mental health (ie, higher self-efficacy, lower kinesiophobia, and pain catastrophizing) and physical activity (ie, higher moderate-to-vigorous physical activity [MVPA], lower sedentary time) would be associated with higher patient-reported hip function.

## METHODS

This cross-sectional study had ethical approval from the West Virginia University (WVU) Institutional Review Board. Individuals with chronic hip pain were recruited from the WVU School of Medicine Physical Therapy Faculty Group Practice from March 2021 through April 2022. To be eligible for the study, patients must have had current hip pain present for a minimum of 3 months. Possible hip conditions included osteoarthritis, femoroacetabular impingement syndrome, acetabular dysplasia, and greater trochanteric pain syndrome. They also had to speak and read fluent English for survey validity. Individuals with a history of surgery to the painful hip or other current spine/lower extremity injuries were excluded. To facilitate recruitment, the treating physical therapist (C.A.M.) notified the study team that there was a patient who met the study criteria. A study team member (K.N.J., D.D., or M.M.) went to the physical therapy clinic, and the physical therapist facilitated a soft hand-off between the patient and the study team member. In a private room, the study team member introduced the study to the patient and answered all questions. If the patient was willing to participate, they signed a consent form and proceeded to complete a series of self-reported questionnaires: 11-item Tampa Scale for Kinesiophobia (TSK-11), Pain Catastrophizing Scale (PCS), Pain Self-Efficacy Questionnaire (PSEQ), and the 12-item International Hip Outcome Tool (iHOT-12). Surveys were housed in a secure REDCap database. Afterward, participants were given an ActiGraph accelerometer (wGT3X-BT) collecting at 60 Hz and were instructed to wear the accelerometer on a waistband centered over the painful hip for the following 7 days, removing it to sleep and shower.

### Survey + Accelerometer Information

#### TSK-11

The TSK-11 is an 11-item questionnaire designed to assess the presence of kinesiophobia (eg, fear of painful movement/reinjury, pain-related anxiety which is hindering participation in physical activity) and is valid within populations experiencing chronic musculoskeletal pain.^18,48^ The TSK-11 is an abbreviated form of the original iteration of the TSK, which features 17 items.^18^ For each of the items on the TSK-11, participants rank the item from a scale of 1 (strongly disagree) to 4 (strongly agree).^18^ The score of each item is summed, and a range of possible scores exists from 11 to 44; higher scores indicating greater kinesiophobia or fear of pain and injury associated with the movement/activity.^18^ This scale has shown to be a brief, reliable, and valid measure of one’s fear of movement as it relates to injury.^43^

#### PSEQ

The PSEQ is a 10-item survey that can be administered to individuals with a chronic musculoskeletal pain disorder to assess their confidence to cope with their pain and complete daily or valued activities despite the pain they are experiencing.^35,50^ For each item on the questionnaire, participants rank the item from 0 (not at all confident) to 6 (completely confident).^50^ The score for each item is combined to yield a possible total score of 0 to 60 with higher scores indicating greater confidence of the individual to complete various activities in spite of their pain.^50^ The questionnaire has demonstrated good reliability, validity, and internal consistency.^5^

#### PCS

The PCS is a 13-item questionnaire that assesses an individual’s level of pain catastrophizing (worst-case thinking, ruminating on and magnifying pain, feeling helpless to decrease their pain). It is valid and reliable in chronic musculoskeletal pain populations.^42^ Each item is scored on a 5-point Likert scale ranged from 0 (never) to 4 (always).^42^ Total scores range from 0 (no catastrophizing) to 52 (highest level of catastrophizing).

#### iHOT-12

The iHOT-12 is a list of 12 questions designed to assess patient-reported hip function and health-related quality of life, or the impact of hip pain on a patient’s life.^14^ Though it was originally designed to be used in young, active individuals, it has been used in a variety of hip-pain populations including in those with hip osteoarthritis.^14,40^ Each item in the iHOT-12 is accompanied by a visual analog scale; patients mark the scale from 0 (significant impairment) to 100 (no problems at all).^14,33,36^ The average of the items is the total iHOT score.^14^ The iHOT-12 is valid, reliable, and responsive to change.^14^

#### ActiGraph wGT3X-BT Accelerometer

The use of accelerometers in research is advantageous for the ability to continuously record and quantify data related to the wearer’s physical activity status.^27^ A widely used accelerometer for monitoring a subject’s physical activity is the ActiGraph wGT3X-BT,^2,6,10,17,27,39^ which is capable of recording 12 different biometric measures related to a subject’s physical activity. In this study, physical activity measures included (1) MVPA, (2) sedentary time, and (3) step count, which was reported as a descriptive variable (average steps per day), though it was not included in the regression model. Participants were instructed to wear the ActiGraph accelerometer collecting at 60 Hz on a waistband centered over their painful hip for 7 consecutive days, removing it only to shower or sleep. After 7 days, accelerometry data were exported using the ActiLife 6 software (ActiGraph, LLC, Pensacola, FL) and the *Freedson Adult VM3 2011* cutoffs were used to calculate physical activity measures.^41^ Four of the 7 days (the first 3 weekdays and 1 weekend day with 10-12 hours of wear time) were used for analysis. The STrengthening the Reporting of OBservational studies in Epidemiology (STROBE) guidelines were used to ensure a thorough and complete reporting of research conducted in this study.^7^

### Statistical Analysis

Parametric assumptions of normality were evaluated for the primary outcome of interest, iHOT-12 scores. Of the 7 days of ActiGraph wear time, 4 days (3 weekdays, 1 weekend day) were analyzed. Physical activity data were exported into the ActiLife software, and step count (average steps per day), sedentary time (average minutes per day), and MVPA (average minutes per day) were exported. Bivariate relationships between independent variables (MVPA, sedentary time, PSEQ, PCS, and TSK scores) and the dependent variable (iHOT-12 scores) were assessed using Pearson product-moment correlations. We took a data-driven approach to candidate variable selection for the linear regression. Linearity is an assumption of performing a regression; therefore, any independent variables that did not have a significant relationship (*P*≤.05) with the dependent variable were not included in the linear regression model as they were unlikely to contribute significantly to the model given their lack of association with the primary outcome. Collinearity between independent variables was also assessed using Pearson product-moment correlations. Some collinearity between psychological measures was expected; however, if correlation coefficients exceeded 0.50, one of the psychological measures was excluded from the linear regression model.

Descriptive statistics were used to summarize demographic, physical activity, and psychological variables of interest. Variables of interest were compared between diagnosis groups using a 1-way repeated-measures analysis of variance. If a significant interaction was identified, post hoc *t* tests were used to identify which groups differed. Predictors of patient-reported hip function (iHOT-12 scores) were assessed using a linear regression with forward variable selection. All analyses were performed using IBM SPSS Statistics (Version 28), and significance level was set as α ≤ 0.05.

## RESULTS

Forty-five participants were enrolled in the study. Two participants had insufficient wear time of the ActiGraph accelerometer, 1 participant reported hip pain for less than 3 months, and 1 participant reported a previous hip surgery, so they were excluded from the analysis. The final analysis included the remaining 41 participants. Intra-articular nonarthritic hip conditions were the most common diagnoses (53.7% of participants) (FIGURE). Diagnosis groups did not differ in terms of sex, BMI, physical activity, psychological measures, or patient-reported function (*P*≥.09; TABLE 1). However, participants with hip osteoarthritis were older than participants with intra-articular nonarthritic hip conditions and extra-articular hip conditions (*P*<.001).

Sedentary time was not related with iHOT-12 scores (r = −0.18, *P* = .26, TABLE 2) and, therefore, was not included in the linear regression. PCS was colinear with both TSK (r = 0.50, *P*<.001) and PSEQ (r = −0.69, *P*<.001) and, therefore, was not included in the model. TSK and PSEQ scores were related (r = −0.39, *P* = .01); however, this collinearity was expected and acceptable for both variables to contribute to the model. TABLE 2 includes a correlation table for all variables of interest. The final linear regression model examined the relationship of MVPA, TSK-11 scores, and PSEQ scores with iHOT-12 scores. A significant model explained 43% of the variance in iHOT-12 scores. However, after adjusting for the number of predictors, the adjusted r squared value was 38% (*P*<.001) (TABLE 3). The coefficients of PSEQ scores, MVPA, and TSK-11 scores each significantly contributed to the model. PSEQ scores explained 22% of the variance in iHOT-12 scores (*P* = .01). MVPA contributed an additional 14% (*P* = .01) and TSK-11 scores an additional 7% (*P* = .04).

## DISCUSSION

This study assessed the associations between kinesiophobia, pain catastrophizing, and pain self-efficacy with physical activity and patient-reported hip function in people with chronic hip pain attending physical therapy. We hypothesized that higher levels of pain self-efficacy and lower levels of kinesiophobia and pain catastrophizing, and better physical activity levels (higher MVPA and lower sedentary time) would be associated with higher patient-reported hip function. Our hypothesis was partially supported, as the results demonstrated that together, pain self-efficacy, kinesiophobia, and MVPA explained 38% of the variance in patient-reported function. Pain self-efficacy was most strongly associated with patient-reported function, explaining nearly a quarter (22%) of the variance in iHOT-12 scores.

There are a large number of studies supporting the importance of considering mental health in the treatment of individuals with chronic musculoskeletal pain in orthopedic literature. Multiple systematic reviews have highlighted the impact of psychological factors and mental health disorders (eg, anxiety and depression) on pain and patient-reported function following hip arthroscopy in individuals with nonarthritic hip disorders.^4,15^ Additional work in this population has identified moderate to strong relationships between pain self-efficacy, pain catastrophizing, and kinesiophobia, and pain and patient-reported function both at baseline and post hip arthroscopy.^21,22^ Similar work has been done in hip osteoarthritis where preoperative mental health has been strongly correlated with patient-reported pain and function 1 year following total hip arthroplasty, and kinesiophobia has been related to objective measures of function (eg, dynamic balance) in patients with greater trochanteric pain syndrome.^11,19^

MVPA was also significantly associated with patient-reported function, contributing 14% of the variance in the model. Increasing physical activity and movement through pacing is a primary therapeutic goal for patients with chronic musculoskeletal pain. To date, objective physical activity measures such as those collected in this study had yet to be examined in combination with mental health to holistically evaluate the combined or individual contributions to patient-reported function in individuals with chronic hip pain. This study helps to increase the body of knowledge in this field and highlights the combined roles that pain self-efficacy and MVPA may play for improving patient-reported hip function.

On the other hand, sedentary time was not related to patient-reported function, suggesting that the intensity level of physical activity may have important implications for patient-reported improvement and overall joint health. Clinicians should encourage their patients to build up to a minimum of moderate physical activity (examples: brisk walking, swimming).^31^ This is consistent with previous literature that supports exercise and physical activity as a means to improve pain severity, physical function, and quality of life.^9,13^

### Clinical Implications

Limitations with a cross-sectional design prevent us from determining causation of the associations found in this study. However, the findings are supported theoretically by the FAM of chronic pain,^49^ which highlights the important mediating role of self-efficacy in the cycle of fear-based thinking (catastrophizing or kinesiophobia) and disability. To begin understanding the role of psychological factors such as pain self-efficacy on clinical outcomes, including patient-reported function, clinicians may integrate a screening process for psychological factors into their clinical practice. In patients presenting to physical therapy for chronic hip pain, psychological patient-reported outcome measures can be given at the initial session to provide a baseline.^1^ Such measures should be carefully selected to minimize patient burden. Given the available evidence, and findings from this study, PSEQ (10 questions) and TSK-11 (11 questions) may be warranted. Screening for psychological response to pain is a key feature of psychologically informed practice, which involves integration of psychological skills into traditional rehabilitation (eg, movement retraining, strength training, physical activity pacing, etc). Theoretically, getting people moving well *and* improving their relationship with pain (pain-related thoughts, feelings, coping strategies) has the greatest potential to improve patient-reported function and quality of life.

The sum of findings from this study and previous literature displays the interconnectedness of psychological factors and physical activity in a patient’s perception of their hip function. In the treatment of individuals with chronic hip pain, it may be prudent to look at more than just biological etiologies for the management of their condition. Instead using a biopsychosocial approach to patient evaluation and rehabilitation through a model of psychologically informed practice.^1,24^

### Future Research

This study prompts multiple promising avenues for future research to deepen our understanding of how to effectively help patients with chronic hip pain. For example, future work may examine pain self-efficacy as a potential mechanism to improving hip function in patients presenting to physical therapy for chronic hip pain. While this study highlights an association between pain self-efficacy and patient-reported hip function, future work should examine this mechanistically. If self-efficacy mediates the relationship between pain and function in patients with chronic hip pain, effective interventions to improve self-efficacy would be warranted. A recent meta-analysis provided only low-quality evidence for the efficacy of current interventions to improve pain self-efficacy in people with chronic musculoskeletal pain.^32^ Additional research into efficacious interventions to improve self-efficacy are necessary. Larger-scale studies with a more diverse population are also warranted to enhance the generalizability of these findings. By addressing these research gaps, we may eventually develop novel treatment approaches that may better improve the lives of individuals living with chronic hip pain.

### Limitations

The sample size was small, the design was cross-sectional, and 70% of the participants were female. Though this is consistent with the sex distribution typically seen in many chronic hip pain conditions, this should be considered, and caution should be used when extrapolating these findings. Additionally, participants in this study included a variety of pain-generating hip pathologies, rather than a single focal pathology. Although not disease specific, the results of this study can support the idea that individuals experiencing chronic hip pain may potentially see an improvement in their self-identified hip function if the levels of physical activity and mental health are addressed holistically in physical therapy. Lastly, this study’s participants were identified from a physical therapy clinic that treated many of the faculty and staff of an academic hospital. As such, measured physical activity levels may be specific to the demands of a working in a hospital setting.

## CONCLUSIONS

Higher pain self-efficacy, lower kinesiophobia, and increased MVPA were associated with higher patient-reported function in people with chronic hip pain of multiple etiologies. Pain self-efficacy was most strongly associated with patient-reported function.

## Figures and Tables

**FIGURE:**
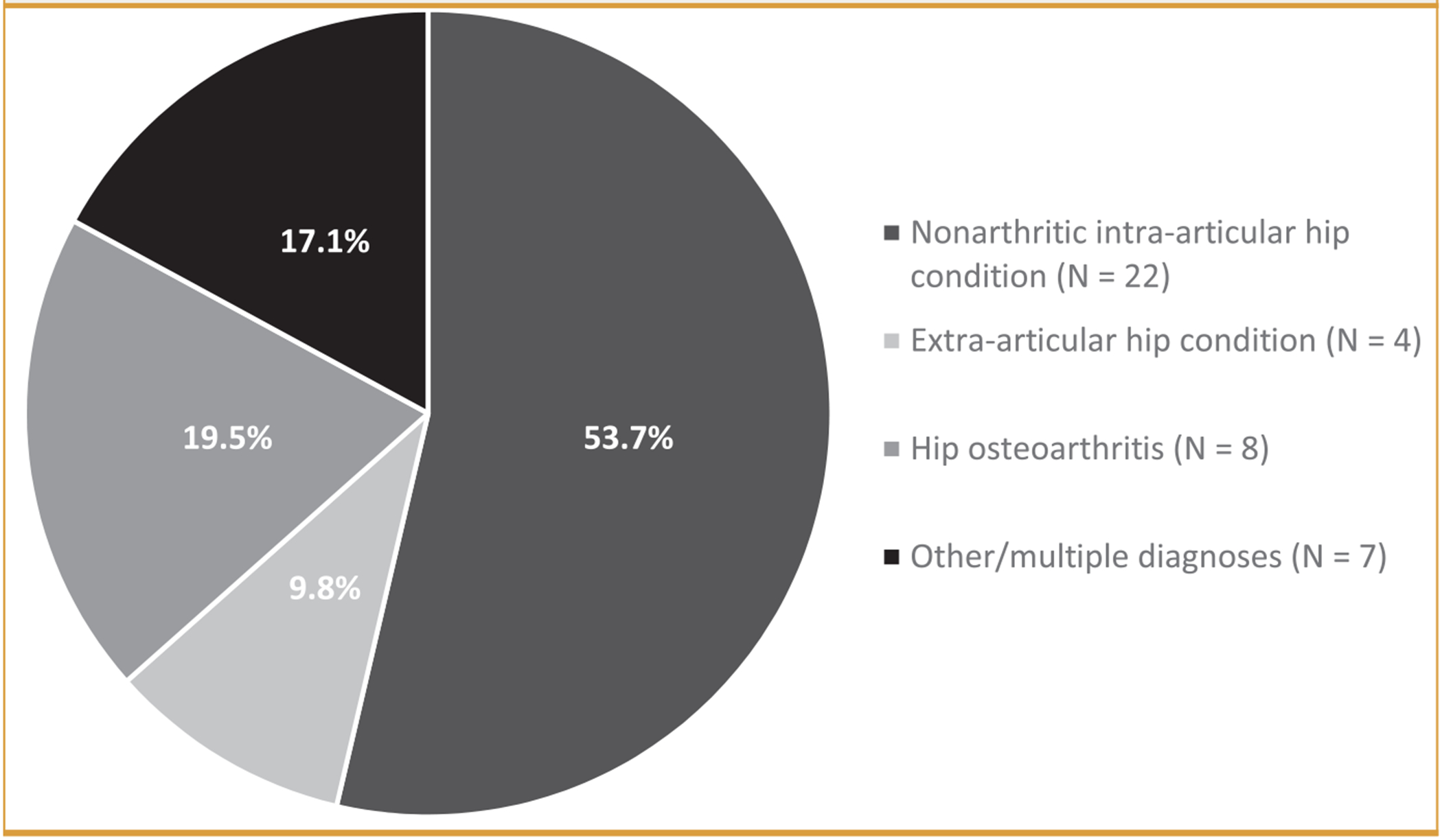
Clinical diagnosis of underlying chronic hip pathology attributed to hip pain experienced by participants (N = 41).

**TABLE 1:** Summary of Demographic, Physical Activity, and Psychological Variables of Interest (VOIs) for All Participants, Including Comparisons Between Diagnosis Groups

VOI	All Participants	Intra-articular, Nonarthritic	Extra-articular	Osteoarthritis	Other/Multiple Diagnoses	*P* Value
N (%)	41	22 (53.7%)	4 (9.8%)	8 (19.5%)	7 (17.1%)	-
Sex (F/M)	29/12	15/7	3/1	4/4	7//0	.20
Age	40.5 ± 14.0	33.0 ± 9.7	48.5 ± 8.5	59.8 ± 8.3	37.4 ± 10.6	**<.001**
BMI	26.7 ± 7.8	24.6 ± 6.9	31.9 ± 6.2	27.1 ± 4.7	29.8 ± 12.2	.22
Symptom duration (months)	23.7 ± 37.2	21.8 ± 24.6	15.8 ± 14.2	14.0 ± 19.8	45.0 ± 76.4	.39
Sedentary time (min/day)	336.9 ± 118.3	330.1 ± 141.2	359.0 ± 133.4	334.6 ± 72.8	348.6 ± 87.5	.97
MVPA (min/day)	34.5 ± 23.8	36.4 ± 25.6	49.2 ± 40.8	26.3 ± 12.1	29.4 ± 14.3	.42
Step count (steps/day)	5844.5 ± 2658.8	5441.0 ± 3121.2	8475.2 ± 3005.5	5810.2 ± 1092.5	5648.4 ± 1365.3	.22
TSK	26.7 ± 6.0	25.4 ± 5.6	30.5 ± 6.5	27.5 ± 7.4	27.9 ± 5.1	.38
PCS	15.5 ± 10.5	13.6 ± 10.7	21.5 ± 3.1	14.9 ± 11.4	18.6 ± 11.3	.46
PSEQ	41.6 ± 10.6	45.0 ± 9.1	37.5 ± 9.3	40.8 ± 11.0	34.1 ± 12.2	.09
iHOT-12	46.1 ± 19.2	46.6 ± 18.2	46.8 ± 25.6	48.5 ± 20.6	41.2 ± 20.8	.90

Abbreviations: BMI, body mass index; iHOT-12, 12-item International Hip Outcome Tool; MVPA, moderate-to-vigorous physical activity; PCS, Pain Catastrophizing Scale; PSEQ, Pain Self-Efficacy Questionnaire; TSK, Tampa Scale for Kinesiophobia.

**TABLE 2:** Pearson Product-Moment Correlations Between All Variables of Interest^a^

	MVPA (min/day)	Sedentary Time (min/day)	TSK-11	PSEQ	PCS	iHOT-12
MVPA	-					
Sedentary Time	−0.01 (0.94)	-				
TSK-11	−0.10 (0.53)	0.16 (0.31)	-			
PSEQ	−0.01 (0.95)	**−0.33 (0.04)**	**−0.39 (0.01)**	-		
PCS	0.08 (0.60)	0.17 (0.28)	**0.50 (*P*<.001)** ^b^	**−0.69 (*P*<.001)** ^b^	-	
iHOT-12	**0.37 (0.02)**	−0.18 (0.26)^b^	**−0.47 (0.002)**	**0.47 (0.002)**	**−0.31 (0.05)**	-

Bold indicates statistically significant P≤.05.

Abbreviations: iHOT-12, 12-item International Hip Outcome Tool; MVPA, moderate-to-vigorous physical activity; PCS, Pain Catastrophizing Scale; PSEQ, Pain Self-Efficacy Questionnaire; TSK-11, 11-item Tampa Scale for Kinesiophobia.

a Reported as Pearson’s r (P value).

b Indicates excluded from the linear regression model: Sedentary time was excluded from the model due to no significant correlation with iHOT-12, and PCS was excluded from the model due to collinearity (r ≥ 0.50) with PSEQ and TSK-11.

**TABLE 3:** Linear Regression Results for 12-Item International Hip Outcome Tool (iHOT-12) Scores in 41 Individuals With Chronic Hip Pain

Predictor	R Square	Adjusted R Square	R Square Change	*B* (Standardized Coefficient)	*P* Value
PSEQ score	0.22	0.20	0.22	0.36	.01
MVPA (min/day)	0.36	0.32	0.14	0.34	.01
TSK-11 score	0.43	0.38	0.07	−0.29	.04
Final Model					
Constant		R Square	Adjusted R Square	Standard Error	*P* Value
34.59		0.43	0.38	15.08	<.001

Abbreviations: MVPA, moderate-to-vigorous physical activity; PSEQ, Pain Self-Efficacy Questionnaire; TSK-11, 11-item Tampa Scale for Kinesiophobia.

## Data Availability

De-identified individual participant data (physical activity/accelerometry and patient-reported outcome measures) that underlie the results reported in this manuscript will be available by reasonable request. Requests must be for research purposes and will be provided under a data sharing agreement. To make a request, please contact the senior author, Dr Kate Jochimsen at kjochimsen@mgh.harvard.edu.

## KEY POINTS

### FINDINGS:

Higher pain self-efficacy, lower kinesiophobia, and increased moderate-to-vigorous physical activity were associated with higher patient-reported function.

### IMPLICATIONS:

Clinicians may consider assessing pain self-efficacy when treating people with chronic hip pain.

### CAUTION:

This cross-sectional study had a small sample size and 70% of the participants were female, which may limit the generalizability of findings.
